# Changes in parenting strategies after a young person’s self-harm: a qualitative study

**DOI:** 10.1186/s13034-016-0110-y

**Published:** 2016-07-02

**Authors:** Anne E. Ferrey, Nicholas D. Hughes, Sue Simkin, Louise Locock, Anne Stewart, Navneet Kapur, David Gunnell, Keith Hawton

**Affiliations:** University Department of Psychiatry, Centre for Suicide Research, University of Oxford, Oxford, UK; Oxford Health NHS Foundation Trust, Warneford Hospital, Oxford, OX3 7JX UK; School of Healthcare, University of Leeds, Leeds, UK; Health Experiences Research Group, Nuffield Department of Primary Care Health Sciences, University of Oxford, Oxford, UK; NIHR Oxford Biomedical Research Centre, Oxford, UK; Central Oxon CAMHS, Oxford Health NHS Foundation Trust, Oxford, UK; Centre for Suicide Prevention, University of Manchester and Manchester Mental Health and Social Care Trust, Manchester, UK; School of Social and Community Medicine, University of Bristol, Bristol, UK

**Keywords:** Parenting, Self-harm, Parents, Mental health, Adolescence

## Abstract

**Background:**

When faced with the discovery of their child’s self-harm, mothers and fathers may re-evaluate their parenting strategies. This can include changes to the amount of support they provide their child and changes to the degree to which they control and monitor their child.

**Methods:**

We conducted an in-depth qualitative study with 37 parents of young people who had self-harmed in which we explored how and why their parenting changed after the discovery of self-harm.

**Results:**

Early on, parents often found themselves “walking on eggshells” so as not to upset their child, but later they felt more able to take some control. Parents’ reactions to the self-harm often depended on how they conceptualised it: as part of adolescence, as a mental health issue or as “naughty behaviour”. Parenting of other children in the family could also be affected, with parents worrying about less of their time being available for siblings. Many parents developed specific strategies they felt helped them to be more effective parents, such as learning to avoid blaming themselves or their child for the self-harm and developing new ways to communicate with their child. Parents were generally eager to pass their knowledge on to other people in the same situation.

**Conclusions:**

Parents reported changes in their parenting behaviours after the discovery of a child’s self-harm. Professionals involved in the care of young people who self-harm might use this information in supporting and advising parents.

## Background

Self-harm (intentional self-injury or self-poisoning, regardless of motive) is relatively common in the UK and Ireland, with an estimated 10–15 % of young people reporting having self-harmed in the past, and 9 % reporting self-harm in the last year [1, 2]. There is considerable evidence for a link between a young person’s relationship with their parents and self-harm [3]. Childhood abuse or neglect is consistently reported as a risk factor for self-harm [4–7] but less extreme family factors such as difficult family relationships [8], low parental care [9], and fear or alienation in the parent–child relationship [10, 11] have also been linked to self-harm. Indeed, young people commonly report difficulties with their parents and family as a reason for self-harm [12], although self-harm can also occur for other reasons, such as difficult peer relationships. Parents’ perceptions of family functioning are reported to be more positive than those of their children, and a large proportion of parents are unaware that their child has been self-harming [3, 13–15]. The discovery of self-harm therefore comes as a shock to many parents. This may lead to feelings of confusion, guilt and worry that they may have contributed to this behaviour [16–20] which may in turn alter their behaviour towards their children. Self-harm in adolescents has been linked to different styles of parenting [15]. To date, very few studies have focussed on how being a parent of a child who is self-harming affects their parenting behaviour, both with regard to the child and any siblings.

Many factors influence parenting, including attachment style [21] and parenting style (e.g., authoritarian, authoritative or permissive [22]), with secure attachment and authoritative parenting styles (e.g., strict but loving) generally associated with better child outcomes [23]. However, when faced with a crisis such as the discovery of self-harm [14], parents may adapt their parenting behaviours. This can include changes to the relative levels of support, control and monitoring [24] of their child and changes in communication with the child. Strategies that emphasise supportiveness include increased praise, hugging or encouragement; those that emphasise control include use of punishment or emotional control to constrain a child’s behaviour; while monitoring relates to maintaining knowledge of a child’s whereabouts, activities and friends [25].

### The current study

We conducted an in-depth qualitative study with parents of young people who self-harmed. We explored how the discovery of a child’s self-harm affects parenting behaviour, including working with their child’s other parent(s), and parenting the child’s siblings. Parents also reflected on specific techniques that they found to be helpful in parenting their child, and which might help other parents.

## Methods

### Sample and recruitment

Thirty-seven parents of 35 young people aged under 25 years who had self-harmed (including two parent pairs) were included in the study. Participants were included regardless of how they interpreted the motive(s) of their child—for example, the level of suicidal intent. A further two people, who did not differ in demographic terms from those included, were interviewed but later withdrew from the study. One person whose husband self-harmed and one person whose sister self-harmed were not included in this analysis. We used a variety of recruitment methods: mental health charities, support groups, clinicians, advertisements, social media, personal contacts and snowballing through existing contacts. Potential participants received an introductory letter, an information sheet and a form to return if they wished to participate. They were encouraged to ask questions about the study, and all interviews were arranged in locations of their choosing.

We sought a maximum variation purposive sample [26, 27] in order to capture a wide range of experiences. We aimed for variation across demographic characteristics including gender, ethnicity (while recognising the difficulties in recruitment that this can present [28]), and geographical location.

### Data generation and analysis

Participants were interviewed between August 2012 and October 2013. Interviews, which took 1.5 h on average, were video- or audio-recorded and consisted of an open-ended section in which the participant explained their experiences of caring for a young person who self-harmed, followed by semi-structured prompts based on topic areas identified through a literature search and suggestions from the project’s Advisory Panel (which included parents, researchers and clinicians). Participants were interviewed by NH or SS, both experienced interviewers.

The interviews were transcribed verbatim from audiotapes by professional transcribers and checked by the researchers. Participants could remove any part of the interview before giving their written consent for the content to be used in research and for publication on a website [29], where a summary of the overall interview findings is available. Final transcripts were uploaded to NVivo 9 for initial coding by NH and SS. A coding framework of both anticipated and emergent themes was developed using constant comparison techniques. Data were assigned to categories using the NVivo ‘node’ function, based on close reading and interpretation of the interview transcripts. Coding reports were generated and used for an initial overarching thematic analysis [30]. Broad themes were then identified based on the summary of all the issues raised by participants on particular topics. Two researchers (NH & SS) conducted this analysis independently and resolved any discrepancies or differences of interpretation through discussion. A more focused analysis on the themes relating to the impact of self-harm on parenting strategies was then conducted by AF using QDA Miner Lite 4 software. Themes were derived from a combination of previous literature and clinical experience of the research team (anticipated themes), and paying close attention to the detail of parents’ accounts (emergent themes). Coded segments of data on topics related to parents’ descriptions of parenting strategies were analysed [30] to identify the broader themes Theoretically, our analysis is informed by symbolic interactionism, which suggests that people act towards things (including events and experiences) based on the meaning those things have for them, and that these meanings are derived from social interaction and modified through interpretation [31]. Basing our analysis on this approach means that while the physical reality, the ‘facts’, of self-harm is one aspect of the phenomenon, the focus of interest is in the interpersonal and social realm.

Participants gave informed written consent before their interview. Pseudonyms were assigned to all participants to ensure confidentiality and anonymity. The study was approved for national recruitment by the Berkshire Research Ethics Committee (09/H0505/66).

## Results

The study participants were from England, Scotland or Wales. Twenty-nine of the young people who self-harmed were daughters and six were sons (Table 1). Self-harm in adolescents in the general population is far more common in girls than boys [1]. Average age at the time of their first episode of self-harm was 15.1 years, with most aged under 16 years. Methods of self-harm primarily included self-cutting and overdoses; other methods included, for example, burning and strangulation. All had engaged in multiple acts of self-harm. Some of the young people had mental health problems, which could include depression, borderline personality traits, anxiety and eating disorders (which parents often saw as a form of self-harm), although not all were formally diagnosed.

**Table 1:** Demographic characteristics of young people who self-harmed and their parents

	Females	Males
	N = 29	N = 6
*Young people*		
Average age started self-harm	13.8 years	16.3 years
Range	9–20 years	9–21 years
Average age at time of interview with parents	18.7 years	22.8 years
Range	14–24 years	17–28 years

	N = 32	N = 5
*Parents*		
Ethnicity	White: 31	White: 5
	Black: 1	

**Fig. 1:**
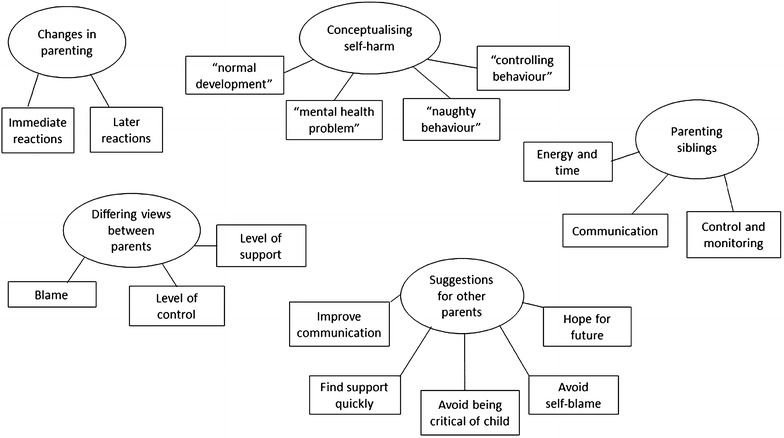
Thematic map showing main themes and subthemes

Overarching themes (Fig. 1) included changes in parenting strategies after the discovery of self-harm, the effect of parents’ conceptions of self-harm on how they parented, the effect of differing views on parenting between parents, parenting siblings and the long-term effects of self-harm on parenting. We also discuss parents’ suggestions for other people in the same situation.

### Changes in parenting

Parents discovered the self-harm in varying ways: some suspected a problem while others were surprised when they were approached by a teacher or friend [20]. Parents’ immediate reactions to self-harm were often highly emotional: regardless of the circumstances of the discovery of self-harm, they described feelings of shock, anger and fear. Elsewhere we have shown that in order to come to terms with self-harm in the family, parents must work through their initial feelings and decide how to handle the changes in their relationship with their child [17]. In some cases this led to immediate and dramatic changes in parenting strategies, such as increasing monitoring or control over their child.

Initially, many parents tried to exert control over the self-harm by, for example, removing access to means. Amber hid her daughter’s blades because “I just needed to do something. I needed to feel that I actually had some control because as a parent you’re programmed to make it all alright and this is something that you can’t make alright.” However, others felt this didn’t help: Janet thought it was “pointless” to try and keep the home free of anything her daughter could use to self-harm.

There was a tendency for parents to keep a closer eye on the child. Nancy was “literally checking [her daughter] every day… making her keep her bedroom door open.” Some parents began to monitor their child in other ways: Judith checked her daughter’s phone to see “what was going on in her life” and Sally checked both her daughter’s phone and diary. “I do it when she’s sleeping, [to] see if there any information I need to know.”

Other parents believed that over-monitoring their child was counterproductive. Theresa characterised this as “not overreacting… one of the most challenging aspects of the whole thing was not to overreact.” Paul worried that constantly watching over and questioning his daughter would “force her into being more wound up.”

Unsurprisingly, most parents also tried to increase supportive parenting strategies. Shannon would “give [her daughter] a cuddle”. Judith read that thinking of distractions might help her daughter avoid self-harm, so she made a list of ideas. “Go and walk the dog. Go and phone a friend. Just come down and see me if you need to cry.” Janet and her daughter worked together to identify circumstances that often preceded self-harm and develop specific coping strategies, such as avoiding over-tiredness and discussing a specific plan for the next day. Sally said that giving her daughter extra cuddles had been “quite therapeutic for her… and… also [reduced] the thoughts [about self-harm] and carrying them out because she knows I’m there for her.” Shannon felt that her own experience of mental health problems in the past allowed her to provide emotional support to her daughter.

Several parents coped by adopting a very matter-of-fact manner. Louise “was very practical… afterwards I fell apart but, at the time, I was very together and I just… got the Steristrips out.” Similarly, Amber felt that “practical mode was easier to deal with than emotional mode… so you look after the cuts because that’s the easy bit.”

Initially, many parents reported “walking on eggshells” around their child in order to avoid upsetting them or triggering another episode of self-harm. Amber felt she “couldn’t even have a normal row with my daughter because I was so scared… she’d get upset and go upstairs and self-harm.” This could change the balance of power in the relationship. Nancy’s daughter “tried it on a bit at first and she knew she was getting away with things she wouldn’t usually get away with.” However, over time many parents learned to be more assertive with their child: Nancy eventually “moved on from thinking [that] I’ve got to let her have her own way” and Amber felt she had “grown a backbone”.

### Conceptualising self-harm and the impact on parenting

Parents’ decisions about strategies to use after the discovery of self-harm depended to some extent on how they conceptualised their child’s self-harm. When parents considered a child’s behaviour to be normal for their developmental stage, or when parents linked it to mental health problems, relatively more supportive strategies were described. A belief that the self-harm was deliberate “bad” behaviour often led instead to increased monitoring and control of the child.

Several parents associated their child’s behaviour with the normal turmoil of adolescence. Roberta thought her daughter was “going through a phase, because she’s thirteen and thirteen-year-olds are awful” while Judith bemoaned the “lies and all the sneakiness… that came with being a teenager.” Others found it difficult to determine the line between “bad” or “naughty” behaviour that they should curtail, and behaviour which could be attributed to symptoms of a disorder that was not the child’s fault. Jennifer struggled with where to draw this line. “I’m not saying that my older daughter should be excused everything because she’s got a mental illness but… where is the mental illness and where is simply bad behaviour? “Joan believed this could affect how supportive a parent should be. “Sometimes I can be very sympathetic and sometimes I can’t because sometimes I think it is naughty behaviour and sometimes I think it’s mental health behaviour.”

Sometimes parents noticed patterns in self-harming behaviour that might explain their child’s actions, which could affect how supportive they felt they could be. Nadine thought her daughter’s crises tended to happen when the focus of attention was on someone else in the family, while Sally noticed that her daughter’s incidents “happen[ed] at times when… she didn’t want to face a situation.” This could lead to the use of relatively less supportive parenting strategies, such as being stricter.

Some parents reported that their child used the threat of self-harm as an attempt at control. This can be part of a broader pattern in which parents feel manipulated by their children, or that their child is using their self-harm to gain attention or control the family. Joy reported that her daughter was upset by Joy’s relationship with a new partner and that she said she would self-harm if Joy did not end the relationship. Judith’s daughter threatened self-harm if she was not allowed to visit a friend. Christopher was convinced that his son was using his depression and self-harm as an excuse “not to go to school, not to do homework and not to eat the food which is put in front of him… Something which he doesn’t feel like doing, says, ‘Oh, you can’t make me do that… I’ll have an episode.’” Although he didn’t deny his son’s problems, his son’s jokes (“Well, if you don’t give me some nice presents for Christmas, I don’t know what’s going to happen”) made Christopher believe his son was using the threat of self-harm to get his own way.

### Differing views between parents

Under the added pressure of worries about a child’s self-harm, differences in the strategies each parent preferred to use could cause conflict between parents. Theresa felt it was important to acknowledge her son’s feelings, but her husband thought “you should just get on with it… so there were differences in the way that we approached this thing which… caused some conflict.” Nancy said her daughter’s father “blamed me because he’s saying that… I condoned her behaviour.”

Differences could occur in the amount of control parents exerted: Shannon felt she was stricter with her daughter than her ex-husband was. “Her dad will… [let her] get away with a bit more… I can be the firmer hand.” In Isla’s family, the opposite was true. “She was beginning to push boundaries quite a lot and my attitude to bringing up children is vastly different to her father. I’m on the much more relaxed, perhaps too relaxed end of the scale. Her father is much more punitive and strict.”

Sometimes one parent felt that they were more supportive of the child than the other parent. Sian said her husband was “not a very emotionally demonstrative person. He’s not very good on the reassurance and the cuddles [although] … he obviously cares.” Similarly, Susanne said her husband was “not really good with the emotional side of life” and tended to back off when her daughter was upset. Denise’s husband found it difficult to talk to their daughter, and Denise felt like “piggy in the middle” as she tried to facilitate communication between them. In other cases, the child treated parents differently—Amy’s daughter “loves her dad but she won’t open up to him and when she’s in crisis it’s me she comes to” and it was difficult for the father not to feel rejected.

### Parenting of siblings

Parents with other children had the additional burden of balancing the needs of all their children. Because considerable parental energy had to be allocated to the child who was self-harming, siblings could become less of a focus. Jacqueline said, “It is really, really hard on a sibling… it [is] very, very easy in this situation for siblings to get lost, for parental attention to be absolutely on [the self-harming child]”. All of Jennifer’s “energy and focus went on [her] older daughter.”

Communication with siblings could be affected. Parents sometimes deliberately concealed a child’s self-harm or mental health issues, especially from younger siblings or those who were thought to be incapable of understanding. This could be an attempt to protect siblings from being upset. Amy’s family struggled with their decision not to tell her daughter’s siblings about the self-harm. She said,

The information that we gave them, looking back, was just minimal. They knew that she was a bit down and was struggling with things…. I think we did the wrong thing in keeping everything back from them…The younger one, I think she resents the fact that we didn’t tell them what was going on at the outset.

Parents may increase control of siblings or monitor them more closely because they are worried about them “copying” self-harming behaviour, and indeed, in some families more than one child had self-harmed. Rebecca said, “I don’t want [my younger daughter] to think [self-harm] is normal.” Some parents tried to restore balance by “compensating” some of their children with money or gifts. Amy bought her younger children gifts to make up for the amount of time she spent caring for their sister, while Jennifer said, “I gave [my other child] money because I was ashamed as I didn’t give her any attention.”

### Longer-term effects on parenting

Most parents said their parenting strategies changed over time. In part, this had to do with testing different coping strategies and discovering by trial and error what helped their child, often aided by their own research and sometimes by speaking to other people (whether parents or clinicians) with experience related to self-harm.

Over the long term, particularly when a child did not seem to be improving, some parents reported becoming worn down. With each succeeding crisis parents were more likely to react with exhaustion rather than panic. In Martha’s words, “initially, I was horrified and very distressed and now I just feel very sad really and sometimes impatient.” These feelings of annoyance or impatience were common when self-harming behaviour continued for a long period of time. Nadine said “her self-harming makes me cross a lot. It makes me angry and upset but mostly it makes me cross. It makes me cross that she does that to herself.”

Parents’ thoughts about the future reflected their expectations about letting their child go. Amber said, “There’s part of you that wants to keep that person so close to you. You just want to… keep them safe… but you can’t because… they have to grow up. They’ve got to make their way.”

Parents struggled with supporting their child while also maintaining their own life. Joy “told her [daughter] I will love her, I will always be there for her… but I need my own life as well because one day, she’s going to have her own life and I won’t have one. I’ll be just left.” Almost 10 years after discovering her daughter’s self-harm, Amber was “only just now getting a proper life back where I will do things because I want to do them, not because it fits in with my daughter.”

### Suggestions for other parents

Given the parenting requirements associated with self-harm—managing a child’s distress, responding to inappropriate behaviour, avoiding feeling controlled by the child—parents often developed specific strategies. Some of these had been suggested by outside agencies (e.g., clinicians) while others were based on parent re-evaluating their previous strategies or discussion with the child or family about how to modify their parenting.

Parents suggested trying to improve communication with the child, even if they did not want to talk face to face. A helpful nurse suggested that Louise’s daughter could send her a blank text when she felt upset but couldn’t talk about it. “When somebody is feeling so miserable that they can’t even talk about it, rather than reaching for something to harm themselves with, to reach for their phone.” Susanne had a notebook where her daughter could write things down that she did not want to talk about and slip it under her mother’s door.

Parents had a wealth of advice for other parents who discovered that a child is self-harming. A common theme was avoiding being overwhelmed by guilt and shame. Isla said, “It’s about not beating yourself up… it’s not a blaming thing.” Parents, such as Roberta, felt that falling into self-blame was not helpful. “I think people think, ‘Oh, what did I do? I’ve led her [or] I’ve led him to do this.’ And that’s not necessarily the case… it’s not necessarily something you’ve done.” Similarly, parents explained the importance of parental self-care—parents must, in Amber’s words, “be really kind to [them]selves.”

Most parents also recommended finding support, help and information about self-harm as quickly as possible. Jennifer’s tactic was to “Make [a] fuss. Ask for help. Don’t consider waiting for referral for 6 months is okay.” Julian spoke about the importance of finding information. “Inform yourself from absolutely every source you can find. From other parents, from books, from the internet, from research papers, so that… you know what you’re dealing with and that way you will be able to talk to professionals on their own terms and be able to make intelligent decisions about your child’s treatment.” Nadine said, “if there are things out there that you think might help, things like mindfulness… or [cognitive behavioural therapy]… look for it.”

Several parents recommended taking care not to be critical of the child, overreact to the self-harm, or make the child feel guilty. They suggested that instead, parents should be attentive to their child and try to understand them without attempting to control them too much, as this might drive the child away. Shannon said, “on discovering… self-harm, don’t lose your rag and shout and scream at them. You’ll just drive it underground and scare them and upset them.” Rebecca’s advice was similar:

Pushing your way in and saying, ‘But I love you. You can’t do this because I love you’ is probably the worst thing you can do. I’ve found that anyway. And so my advice would be, act immediately… and get professional help. And if you’re able to, take a step back… it’s very provocative for the child to have someone make them feel guilty.

Parents also gave a message of hope to others. Nadine said, “I think my daughter is living, breathing proof that you can find other strategies. There are other strategies out there and I would hope that somebody who self-harms would be fortunate enough to be able to find services.” Parents whose children had not recently self-harmed wanted to remind others that this period of their life would not last forever. Amber said, “I just feel that I’m now the mother of a very normal 22 year old and…, I wouldn’t wish it on my worst enemy… but it’s made us the people that we are now.”

## Discussion

Parents’ reports indicated that parenting strategies often changed after the onset of a child’s self-harm. This included increased or decreased support, control, and monitoring of the child, which may either be deliberate or occur naturally as parents try different strategies and discover what works. Similar to previous smaller qualitative studies with parents [16, 32], the discovery of a young person’s self-harm often lead to an increase in monitoring, including looking through diaries and phone messages. This could be a way to try and manage the self-harm and their relationship with their child, although parents’ response to self-harm also depended to some extent on whether they viewed the behaviour as “naughty” or whether they associated it with an adolescent developmental phase or mental health problems.

After the discovery of self-harm, the power structure in the family has been shown to change, with parents becoming fearful of disciplining their child [19]. The parents in our study described similar fears, developing coping strategies by trial and error because they worried that their original approaches to parenting contributed to the onset of their child’s self-harm. Over time, most parents found it was important to set boundaries with their child and acknowledge their own needs as well as their child’s.

We found that parents sometimes disagreed with their co-parents on the best approach to take, with some focussing on emotional support and others on setting limits. This could cause discord in the family. The needs of siblings also had to be taken into account, and sometimes parents found their parenting of their other children changed as a result of one child’s self-harm. Several parents reported getting very frustrated when their child’s behaviour did not seem to improve, but eventually many families had a stage of “letting go” when children moved past self-harming behaviour or went off to further education or work, leaving parents to become more of a distant support. This could be an opportunity for parents to feel they had their “own life back”.

### Implications

One important implication is the need for forums or groups where parents who have experienced a child’s self-harm can share their experiences and advice with other parents. This could be facilitated by clinicians or workers with responsibility for young people. Clinicians can also provide advice about potentially useful strategies for parenting a young person who has self-harmed and provide parents with information about self-harm.

### Strengths and limitations

The study included a relatively large qualitative sample of parents who spoke extensively about their parenting experiences. Most participants were mothers, reflecting the difficulty of recruiting fathers for such research [33]. Participants came from around Great Britain (Table 1). Diversity was limited, with only one participant from a minority ethnic background, reflecting the general difficulty in recruiting ethnic minorities for research on mental health issues [34]. We spoke only to parents and are only able to infer the impact on children and other family members from the parent’s account. Although the interviewer clarified the parents’ meanings during the course of the interview, we did not check the finished themes with parents. However, they had access to an online representation of major research themes.

## Conclusions and future directions

A child’s self-harm is very challenging for parents to cope with. It can have a fundamental effect on parenting strategies, with regard to both the child who is self-harming and other children in the family. This can include increased or decreased support, control, and monitoring. Clinicians and school staff with responsibility for young people should be aware of these findings and do what they can to help parents find strategies that are effective for their child and themselves. This could include being aware of the difficulties when parents do not agree on strategies and the need for help in negotiating an approach to parenting that both parents agree with. School staff may work with the siblings of a young person who has self-harmed if they are in the same school. They may also be able to provide them additional support. Parents also indicated that meeting with others in the same situation would be helpful: this could be in the form of a weekly or monthly meeting facilitated by local services. This could include psychoeducation about the nature of self-harm, and the discussion of possible parenting strategies to manage it. Future research could involve the young person as well as the parent(s) in order to assess young peoples’ experiences of the impact of different parenting strategies and their views on what was helpful or unhelpful, as well as their perceptions of family functioning.

## Declaration

This is a summary of independent research funded by the National Institute for Health Research (NIHR)’s Programme Grants for Applied Research Programme (Grant Reference Number RP-PG-0610-10026). The views expressed are those of the author(s) and not necessarily those of the NHS, the NIHR or the Department of Health. The funding body had no role in the design, collection or interpretation of the data or in writing the manuscript.
